# Characterizing random complex biological media by quantifying ultrasound multiple scattering

**DOI:** 10.3389/facou.2025.1545057

**Published:** 2025-04-08

**Authors:** Omid Yousefian, Azadeh Dashti, Haley Geithner, Yasamin Karbalaeisadegh, Shanshan Yao, John Blackwell, Mir Ali, Stephanie Montgomery, Yong Zhu, Thomas Egan, Marie Muller

**Affiliations:** 1 Mechanical and Aerospace Engineering, North Carolina State University, Raleigh, NC, United States; 2 Surgery, University of North Carolina, Chapel Hill, NC, United States; 3 Biomedical Engineering, North Carolina State University, Raleigh, NC, United States

**Keywords:** ultrasound, tissue characterization, random media, multiple scattering, scattering, quantitative ultrasound

## Abstract

**Introduction:**

In this *in silico*, *in vitro*, and *in vivo* study, we propose metrics for the characterization of highly scattering media using backscattered acoustic waves in the MHz range, for application to the characterization of biological media.

**Methods:**

Multi-element array transducers are used to record the ultrasonic Inter element Response Matrix (IRM) of scattering phantoms and of lung tissue in rodent models of pulmonary fibrosis. The distribution of singular values of the IRM in the frequency domain is then studied to quantify the multiple scattering contribution. Numerical models of scattering media, as well as gelatin-glass bead and polydimethylsiloxane phantoms with different scatterer densities, are used as a first step to demonstrate the proof of concept.

**Results:**

The results show that changes in microstructure of a complex random medium affect parameters associated with the distribution of singular values. Two metrics are proposed: *E*(*X*), which is the expected value of the singular value distribution, and $\lambda_{\max}$, the maximum value of the probability density function of the singular value distribution, i.e., the most represented singular value. After validation of the methods *in silico* and in phantoms, we show that these metrics are relevant to evaluate pulmonary fibrosis in an *in vivo* rodent study on six control rats and eighteen rats with varying degrees of severity of pulmonary fibrosis. In rats, a moderate correlation was found between the severity of pulmonary fibrosis and metrics *E*(*X*) and $\lambda_{\max}$.

**Discussion:**

These results suggest that such parameters could be used as metrics to estimate the amount of multiple scattering in highly heterogeneous media, and that these parameters could contribute to the evaluation of structural changes in lung microstructure.

## 1 Introduction

Wave propagation in random complex media has been a subject of interest in a wide range of fields including optics, electromagnetism, solid state physics and seismology (Lax, 1951; Waterman and Truell, 1961; Tourin et al., 2000). In acoustics, when scatterer density is relatively high, the difference between the acoustical properties of scatterers and the surrounding matrix leads to multiple scattering of acoustic waves. As the wave propagates through the highly scattering medium, the coherent wave becomes more challenging to track. Hence, multiple scattering makes conventional ultrasound imaging based on echolocation extremely challenging. This is especially true in complex biological organs such as bone or lung where pores and alveoli act as scatterers. However, the waves backscattered from complex random media contain rich, useful information on the micro-structure of such media. One could retrieve these microstructural properties through statistical approaches. Due to recent developments in manufacturing of multi-element array transducers, biomedical acoustics has the experimental advantage of offering controllable multi-element arrays of quasi-point-like emitters/receivers. In these transducer configurations, the propagation relationship between two elements of the probe is best described by a propagation matrix, also called the Impulse Response Matrix (IRM), **H**. Thanks to the complexity of highly heterogeneous random media, large amounts of the information regarding the interaction between the wave and the microstructure is contained in this matrix. In other words, once the matrix **H** is formed, *ad hoc* post-processing could enable extracting data related to medium micro-structure.

A number of statistical methods have been used in the fields of acoustics and biomedical ultrasound to characterize random complex media using this matrix. For example, Aubry and Derode (2011) used a matrix formalism to separate the single and multiple scattering contributions to backscattered signals in human soft tissue. Their results showed that multiple scattering is not negligible in breast tissue, and could be exploited to detect microstructural changes. Karbalaeisadegh et al. (2019) estimated the diffusion constant of ultrasonic waves in the MHz range propagated through cortical bone. The diffusion constant was estimated using the propagation matrix in a backscattering configuration. The diffusion constant quantifies the rate of acoustic energy distribution in space, due to scattering. It is correlated to the scattering mean free path, which characterizes the mean distance between scattering events. It is therefore expected to be related to scatter density. Karbalaeisadegh et al. (2019) studied how the changes in pore size and pore density independently affect the diffusion constant. Mohanty et al. used the diffusion constant from multiple scattering of ultrasonic waves to characterize the lung parenchyma for pulmonary fibrosis, pulmonary edema (Mohanty et al., 2017), and to detect the presence of non-scattering inclusions in the lung (Mohanty et al., 2020). Dashti and Lye. extracted backscattering parameters such as the backscatter coefficient, and used envelope statistics to quantify changes in rats lungs due to varying degrees of pulmonary fibrosis (Lye et al., 2021; Dashti et al., 2024).

Prada and Fink (1994), Prada et al. (1996) showed that in the case of point-like scatterers randomly distributed in a homogeneous medium, each scatterer can be associated to one non-zero singular value of the propagation matrix in the frequency domain. Hence, Singular Value Decomposition (SVD) of the propagation matrix can provide useful information regarding scattering patterns occurring in a random medium. Aubry and Derode (2010) applied general results of Random Matrix Theory (RMT) (Tulino and Verdú, 2004) on the SVD of the propagation matrix in a backscattering configuration and established detection criteria in the presence of multiple scattering based on the statistical properties of singular values.

In the present study, we use the distribution of singular values from backscattered signals to extract metrics relevant to the characterization of the micro-structure of complex random media. SVD is estimated using a Probability Density Function (PDF) where two parameters of this PDF are studied: $\lambda_{\max}$, the maximum value of the probability density function of the singular value distribution, and *E*(*X*), the expected value of the singular value distribution. In order to analyze how $\lambda_{\max}$ and *E*(*X*) change as the micro-structure of a complex random medium varies, we begin by generating numerical structures consisting of solid scatterers randomly distributed within a fluid with five different area fractions. For each structure, the distribution of singular values is estimated. We study how $\lambda_{\max}$ and *E*(*X*) change as a function of scatterer area fraction. Next, we address the experimental applicability of this technique to differentiate between low and high scatterer volume fractions in Gelatin-Glass Beads (GGB) phantoms that mimic highly scattering media by using multi-element linear array transducers at different frequencies. Furthermore, we examine if this method is applicable to assess varying scattering microstructures in media with varying speed of sound. In order to test this, phantoms including Barium-Titanate particles embedded in a PDMS matrix are fabricated with five different scatterer mass ratios. The distribution of singular values and speed of sound are measured in each case. Finally, in an *in vivo* study, we investigate whether wall-thickening due to pulmonary fibrosis leads to changes in the scattering behavior of lung tissue by studying how *E*(*X*) and $\lambda_{\max}$, are affected by the severity of pulmonary fibrosis.

## 2 Materials and methods

### 2.1 Random Matrix Theory

In this section the acquisition of an Inter-element Response Matrix (IRM) from a multi-element array transducer and the subsequent SVD is described. Some general results from the RMT (Tulino and Verdú, 2004) and its applicability in analyzing the singular value distribution of IRM acquired from multiple scattering structures are also presented.

An ultrasound transducer array with *N* elements is used in a backscattering configuration, so that both emission and reception can be performed with the same probe. A pulse is emitted from a single element *i* into the medium and the backscattered signals are recorded by all *N* elements. This emission process is repeated for all *N* transmitting elements. The backscattered signals form a 3D propagation matrix **H**, the IRM, consisting of *N* × *N* × *t* elements where *t* is the number of temporal data points or signal length. A time trace $h_{ij}(t)$ recorded in the *i*th row and *j*th column of the matrix **H** corresponds to the signal emitted by element number *i*, propagated into the medium, and received by element number *j*. Because of reciprocity, $h_{ij}(t)$ and $h_{ij}(t)$ are expected to be identical. The signals received after emission from element number *i* (*i* = 1, 2, …, *N*) are time shifted such that the signal corresponding to the time of flight between the surface of the probe and the medium of interest is truncated. By doing so, *t* = 0 corresponds to the signal from the surface of the medium for all time traces. In order to keep a temporal resolution for acoustic measurements when switching to the frequency domain, overlapping time windows of length *T* are applied over each signal according to Equations 1a, b:

(1a) $$H_{ij}(\tau ,t)=H_{ij}(\tau -t)W(t),$$

(1b) $$W\left(t\right)=\left\{\begin{array}{ll} 1, & \text{if}\;t\in \left[-T/2,T/2\right]\\ 0, & \text{otherwise}. \end{array}\right.$$

The time window length is chosen in such a way that each time window contains at least one scattering event. Taking this into account, in both experiments and simulations we assumed T=4*period. The number of time windows is based on the whole duration of the acquired raw signals in phantoms and *in silico*. In rat lungs, the raw signals were cut in time based on the estimated thickness of the lung (about 3 cm deep). Each signal in each time window is then converted into the frequency domain using Fast-Fourier Transform (FFT). The frequency range is limited to the −6 dB pulse bandwidth. This creates a 4D matrix **K**, in which element $k_{ij}(\tau ,f)$ refers to the *f*th frequency in τth time window from the signal emitted by element *i* and received by element *j*. Hence, the **K** matrix has $N\times N\times N_{\tau }\times N_{f}$ elements, where $N_{\tau }$ and $N_{f}$ are the number of time windows and frequencies respectively. The SVD of the matrix **K** yields *N* singular values $\lambda_{i}(\tau ,f)$ for each time window τ and frequency f. Since the random complex media studied here are each one realization of a random process, results from RMT can be applied here. The general results provided by RMT are based on the assumptions that the elements of a random matrix have zero mean and variance 1/*N*. Assuming that $k_{ij}(\tau ,f)$ is a superposition of scattering contributions with a phase uniformly distributed between −π and π, the first condition is met. In order to satisfy the second condition, singular values of **K** are normalized as described in Equation 2:

(2) $$\left({\tilde{\lambda }}_{i}\right)=\frac{\lambda_{i}}{\sqrt{\frac{1}{N}\sum_{p=1}^{N}\lambda_{p}^{2}}}$$

After normalization, one can study the distribution of singular values by forming a histogram $ℋ(\tilde{\lambda })$ of all the singular values taken at each time window and frequency. $ℋ(\tilde{\lambda })$ indicates the number of singular values contained in the same bin as $\tilde{\lambda }$. We can define an estimator of probability density function (PDF) of singular values as described in Equation 3

(3) $$\rho (\tilde{\lambda })=\frac{ℋ(\tilde{\lambda })}{nw}$$

where n denotes the total number of singular values ($n=N\times n_{f}\times n_{\tau }$, where $n_{f}$ is the total number of frequencies at each time window and $n_{\tau }$ is the total number of time windows) and w is the bin width of histogram. Two parameters, described in Equations 4, 5 of this PDF are studied to evaluate how they are affected by micro-structural changes:

(4) $$\lambda_{\max}=\max\left\{\rho \left(\lambda \right)\right\}$$

(5) $$E(X)=\int_{-\infty }^{\infty }x.\rho (x)dx$$

It has been shown (Tulino and Verdú, 2004; Aubry and Derode, 2009) that when multiple scattering dominates in a medium, the distribution of singular values follows a ”Quarter Circle Law” (QCL) derived from RMT as described in Equation 6:

(6) $$\rho_{\mathrm{QCL}}(\lambda )=\frac{\sqrt{4-\lambda^{2}}}{\pi }.$$

In reality the propagation of acoustic waves in a random medium is a combination of both single and multiple scattering. This means that the closer the PDF is to a quarter circle, the more multiple scattering occurs in the random medium. A distribution of singular values closer to a quarter circle, reflecting larger amounts of multiple scattering, should be described by a larger expected value *E*(*X*) and a lower value of $\lambda_{\max}$ than a distribution of singular values obtained from a medium exhibiting less multiple scattering. We therefore propose to use these two metrics to characterize the amount of multiple scattering in complex media.

### 2.2 Numerical simulation

In this section, we numerically study how $\lambda_{\max}$ and *E*(*X*) change as the area fraction of scatterers increases in highly scattering media. We simulate media with two different scatterer material properties to determine whether $\lambda_{\max}$ and *E*(*X*) are capable of differentiating between material properties of scatterers as well.

**FIGURE 1:**
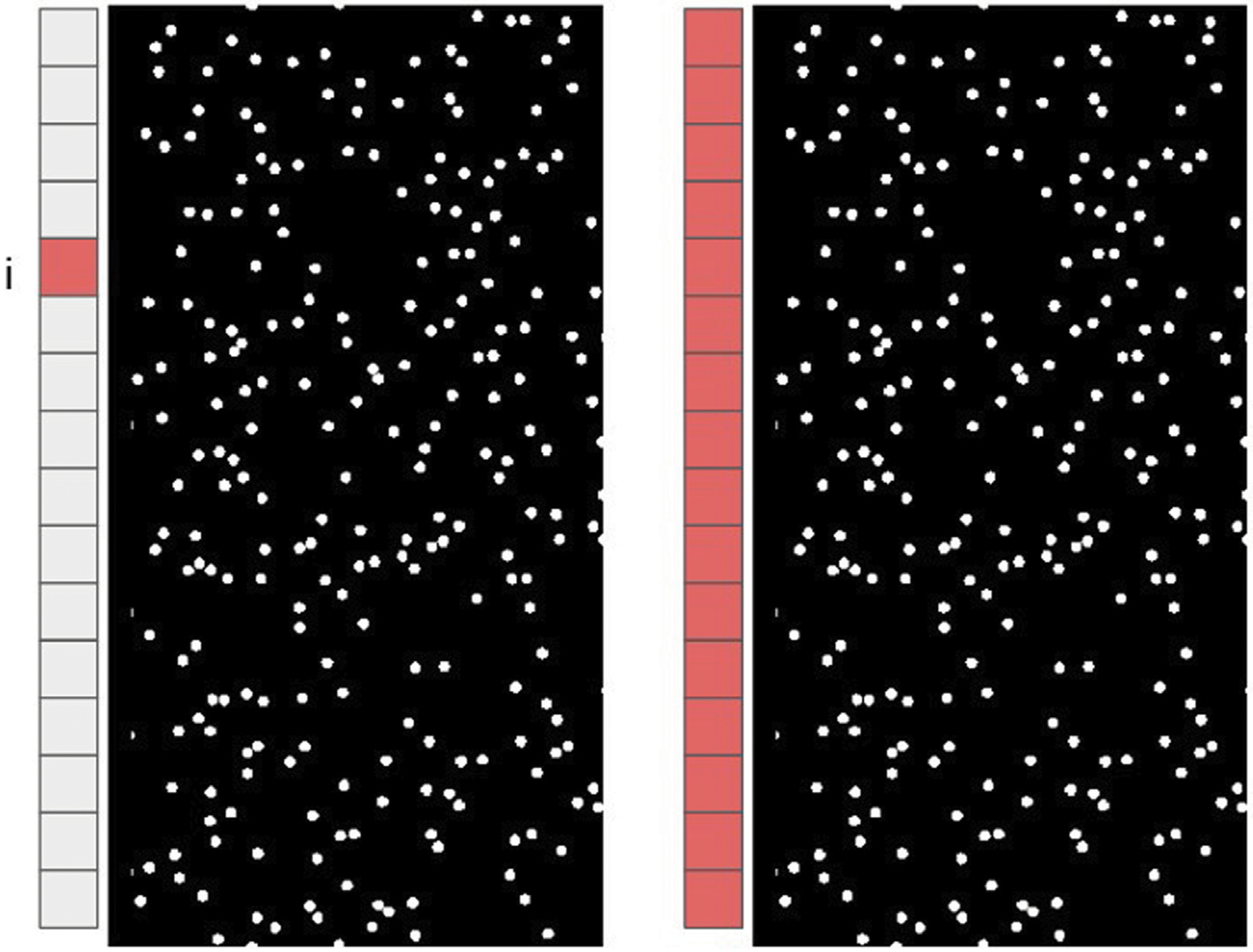
Schematic of emission by element *i* (left) and reception (right) by all the elements.

In order to study how the micro-structure of complex random media might affect the distribution of singular values, 2D numerical structures of mono-disperse circular scatterers made of plastic or aluminium (diameter equal to 500 *μm*) randomly distributed in water are created. Scatterer area fraction $\left(\frac{\mathrm{Scatterer surface area}}{\mathrm{Total surface area}}\right)$ is changed from 5% to 25% in 5% intervals. A multi-element array transducer with 128 elements is simulated, emitting a Gaussian pulse with a central frequency of 5 MHz. An IRM is formed for each numerical structure in backscattering configuration (A schematic is shown in Figure 1). The numerical simulations are performed using the Finite Difference Time-Domain (FDTD) SimSonic freeware (Bossy et al., 2004). The simulation input parameters include material density, and the elastic tensor constants $C_{11},C_{22}$ and $C_{12}$. All simulation parameters are summarized in Tables 1, 2 The material absorption is not taken into account in the simulations.

### 2.3 Phantom fabrication

In this section the preparation and fabrication of the phantom used for the experimental studies are described. Phantoms mimicking complex random media studied in this work are Gelatin-Glass Beads (GGB) and PDMS-Barium Titanate phantoms.

**FIGURE 2:**
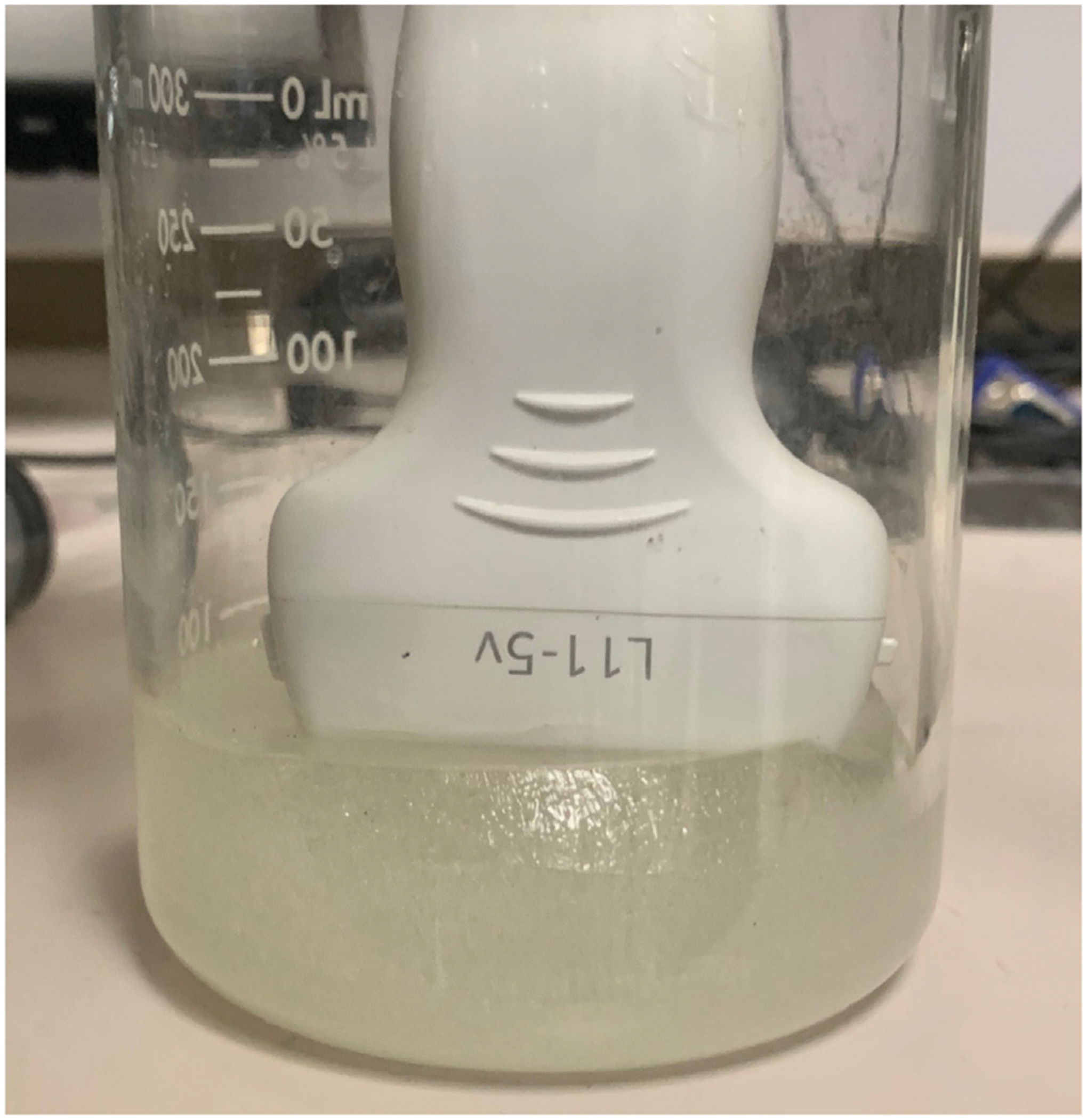
Measuring backscattered signals of Gelatin-Glass bead phantoms using an array transducer.

GGB phantoms are made using a gelatin solution as the homogeneous matrix and glass bead particles acting as acoustical scatterers. First, 5 g of gelatin powder from porcine skin (SIGMA Life Science) are mixed with 100 mL of water (5% by volume). The solution is stirred in a borosil glass container over a hot plate at 70*°C* using magnetic stirrers, in order to create a molten gelatin. Then, 3% by weight (3 g) of glass beads (average diameter = 200 *μm*) is added to the molten gelatin and stirred using magnetic stirrers at room temperature (1.4% volume fraction of glass scatterers). Another, more concentrated phantom is made with 10% by weight glass beads (10 g). For the two phantoms the volume fraction of glass beads are 1.4% and 4.5%, respectively. The stirring is carried out at room temperature for cooling. This is done so that the solution forms a slurry and the gelatin sample becomes viscous enough to ensure no settling of the glass beads was observed. Once the slurry is formed, a few ml of 95% alcohol is added to remove air bubbles and the phantoms are cooled overnight at 4°C before the ultrasound data are acquired (Figure 2).

**FIGURE 3:**
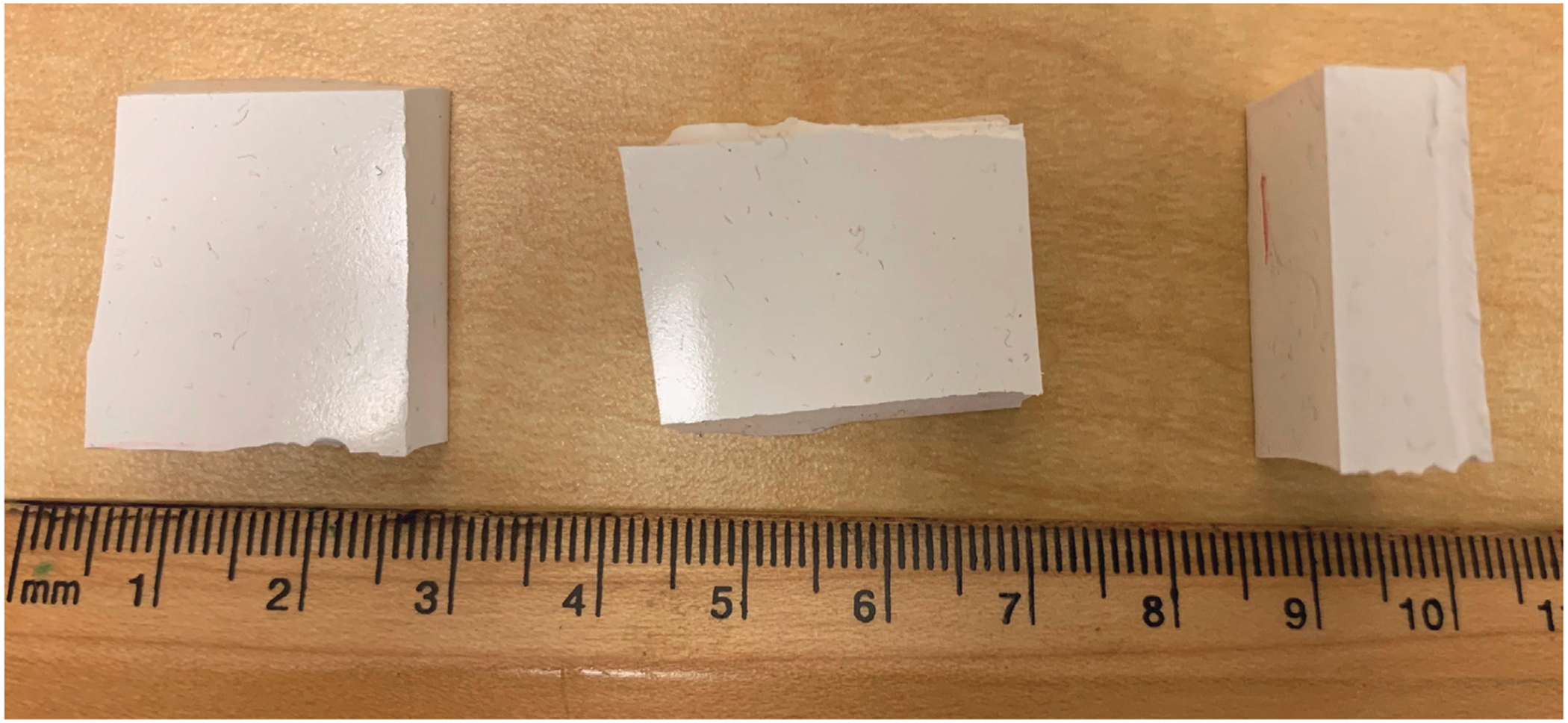
Three samples extracted from the same PDMS-Barium Titanate phantom.

To fabricate the PDMS-Barium Titanate composite, two parts of polydimethylsiloxane (PDMS) (Dow CorningÂ^®^ Sylgard 184) are measured with a base to curing agent ratio of 25:1 in weight and mixed in a plastic beaker (Yao and Zhu, 2014; Yao et al., 2017). BaTiO3 powder (Acros Organics, particle diameter ≈ 0.85 to 1.45 *μm*) is added into the mixture with PDMS to BaTiO3 ratios of 4:1, 2:1, 4:3, 1:1 and 1:2 corresponding to almost 4, 7, 11, 14 and 24 percent volume fraction, respectively. The PDMS-Barium Titanate mixture is stirred thoroughly, degassed in a vacuum chamber for several cycles to remove air bubbles, and cured at 100*°C* for 2 h. The cured PDMS-Barium Titanate composite is cut into the desired shape as shown in Figure 3 before testing.

### 2.4 Ultrasound data acquisition: Phantom study

In this section the distributions of singular values from IRMs obtained in the two types of phantoms are studied. In the first set of GGB phantoms, mimicking highly scattering media, we study if one can assess single and multiple scattering using QCL. We also investigate whether $\lambda_{\max}$ and *E*(*X*) can be used to differentiate between the high and low scatterer volume fractions at different frequencies. Two multi-element array transducers with 5.1 and 7.8 MHz central frequencies are used to explore a large frequency range. In the second set of phantoms, in PDMS-Barium Titanate composites mimicking weakly scattering media, we investigate whether *E*(*X*) and $\lambda_{\max}$ can be used to differentiate between media with different speed of sounds. The reason why we chose to investigate the influence of speed of sound is because it has been shown that micro-structural changes such as increased bone porosity (Wear et al., 2000), malignant lesions in breast tissue (Li et al., 2009) and gastric cancerous tissues (Saijo et al., 1991) affect the ultrasonic speed of sound. In other words, changes in speed of sound in biological tissues can be used for characterization purposes.

The experiments are performed using an L7-4 (ATL, Bothell, WA) and an L11-5v (Verasonics, Kirkland, WA) 128 elements linear array transducers (Figure 2). The configuration of these transducers are summarized in Table 3. The multi-element array transducers are connected to a Verasonics Vantage ultrasound scanner (Verasonics, Kirkland, WA) and configured to emit a Gaussian pulse (centered at 5.1. and 7.8 MHz respectively), element by element. The backscattered signals are then recorded on all of the elements of the array at a sampling frequency of 62.5 *MHz*). In the GGB phantoms, the scatterer size is comparable to the wavelength at 5.1 and 7.8 MHz (wavelength ≈ 0.85 – 1.31 scatterer diameter), suggesting a highly scattering medium. On the other hand, in the PDMS phantoms the scatterers are much smaller than the wavelength at 5.1 and 7.8 MHz (wavelength ≈ 120 – 200 scatterer diameter), suggesting a more weakly scattering medium, but the speed of sound varies significantly with added Barium titanate particles. These two phantom structures should enable us to study the SVD of backscattered IRMs in high and weak scattering regimes, and to study the effect of the speed of sound.

In the PDMS phantoms (Figure 3), using an L7-4 multi-element array transducer, the IRM is acquired at three different locations of each phantom to account for randomness in the scatterer distributions. The L11-5 transducer array was not used with these PDMS phantoms due to the high absorption coefficient of the PDMS matrix, resulting in highly attenuated backscattered signals and very low signal to noise ratios at 7.8 MHz. After IRM acquisition, three samples are cut from each PDMS phantoms (5 × 3 = 15 smaller samples). Assuming that the speed of sound in the phantoms is independent of frequency, using a pair of single element transducers (OLYMPUS) with a central frequency of 1 MHz and 0.5 inch diameter, we measure the speed of sound by submerging the PDMS phantoms in water $\left(c_{s}\right)$ according to a method described in Sasso et al. (2007), which defines the experimentally measured speed of sound as described in Equation 7:

(7) $$c_{s}=\frac{c_{w}d}{d-\Delta tc_{w}},$$

where $c_{w}=1.54\;\mathrm{mm}\cdot \mu s^{-1}$ is the wave velocity in the reference medium (water), *d* is the distance between transducers and Δt is the difference in time of flight with and without the sample. For each scatterer volume fraction, the speed of sound is measured for three samples to take the uncertainty into account.

### 2.5 *In vivo* rodent lungs experiment

#### 2.5.1 Tissue preparation

Thirty-two Sprague Dawley rats are acquired under an approved protocol, as described in Dashti et al. (2024). Six rats with healthy lungs are used as the control group. Pulmonary fibrosis is induced in twenty-six rats, eight of which are treated by Nintedanib. The animal preparation, fibrosis model, and ultrasound data acquisition procedure have been described thoroughly in previous work (Mohanty et al., 2020), but are discussed briefly here. Pulmonary fibrosis is induced by instilling bleomycin into the airway. The rats are sedated and intubated with a 12-gauge catheter. Bleomycin 2 mg/kg is dissolved in 100 *ml* sterile PBS and administered into the trachea. Afterwards, the rats are extubated and left to recover. Ultrasound data acquisitions were performed at least 2 weeks after bleomycin administration.

Prior to ultrasound data acquisition, rats are anesthetized with titrated isoflurane. A sternotomy is carried out to open both pleural spaces and expose both lungs (the intercostal space in rats is too small to accommodate a conventional ultrasound probe). The incision is extended inferiorly into the abdomen to expose the liver, and heparin is administered intrahepatically to prevent clotting after lung removal. A 2 mm (approximately) layer of ultrasound coupling gel is applied directly onto the probe and ultrasound measurements are acquired *in vivo*. After data acquisition, the rats are euthanized by cardiectomy under anesthesia.

#### 2.5.2 *In vivo* ultrasound data acquisition

As in phantoms, ultrasound measurements are obtained using a 128 element linear array transducer (Verasonics L11-4v at a central frequency of 7.8 MHz) connected to a Verasonics Vantage ultrasound scanner using a sampling frequency of 62.5 MHz. The inter-element Response Matrices (IRMs) are recorded for each lung as detailed in section 2.1, and as described in previous work from our group (Mohanty et al., 2020; Lye et al., 2021; Dashti et al., 2024).

#### 2.5.3 Rodent lungs histology measurement

After ultrasound data acquisition, the lung tissue is excised and subjected to inflation-fixation. The lungs samples are submerged in paraformaldehyde for 24–48 h. The lung tissue is then placed in paraffin and cut into sections with 5μm thickness. Lung sections are stained by hematoxylin and eosin (H&E), Sirius red and Masson’s Trichrome and scanned under a ×10 objective magnification using a microscope. The severity of fibrosis is assessed by a veterinary pathologist, assigning a severity score using the modified Ashcroft scale. The Ashcroft score ranks the severity of Masson Trichrome on a scale of 0 (least severe) to 8 (most severe), but none of the slides scored higher than 4. For each animal, the final histology scored was obtained by taking the average of multiple slides (Hubner et al., 2008). The pathologist was blinded to animal groups. Data was re-evaluated after 1 week to check consistency with the first reading.

## 3 Results

### 3.1 Numerical simulations

**FIGURE 4:**
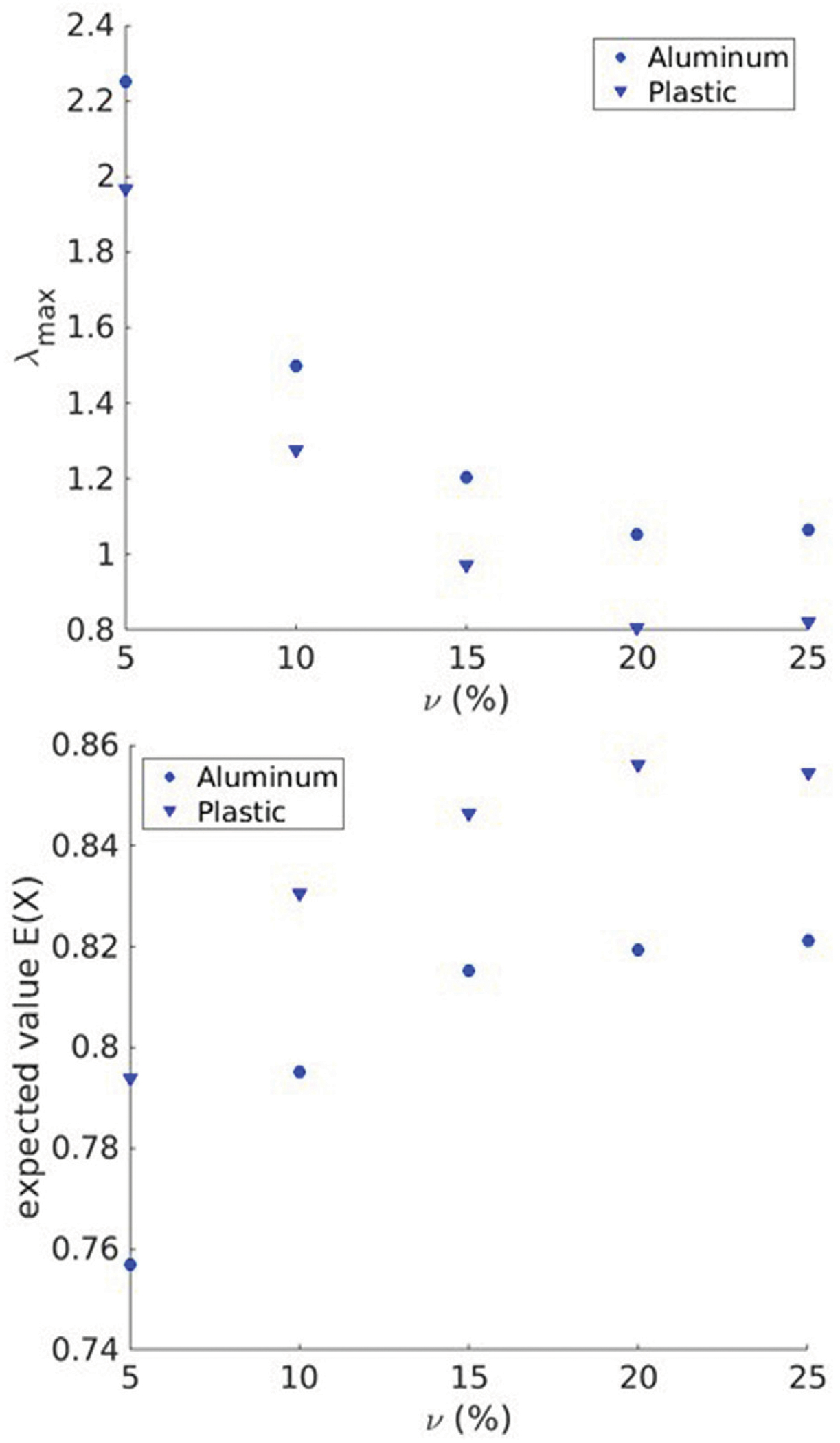
$\lambda_{\max}$ and *E*(*X*) as a function of scatterer area fraction for aluminum and plastic scatterers embedded in water.

The IRMs acquired by FDTD numerical simulations are post-processed to form the PDF for the distribution of singular values according to the method described in section 2.1. For various scatterer area fractions (with scatterers made of aluminum and plastic), $\lambda_{\max}$ and *E*(*X*) were measured. Figure 4 depicts these values vs scatterer area fraction (ν).

As the figures imply, the value of $\lambda_{\max}$ increases with increasing ν up to 20%, after which it reaches a plateau. Additionally, *E*(*X*) decreases with increasing ν up to 20%, after which it reaches a plateau. These numerical simulation results show the potential of this method to differentiate between low and high scatterer area fraction in complex random media. However, beyond a certain scatterer density (20% in this case), multiple scattering dominates and the characteristics of the singular value distribution no longer change significantly. The scatterer’s materials could be distinguished using $\lambda_{\max}$ (10% difference for aluminum and plastic) and *E*(*X*) (5% difference for aluminum and plastic).

### 3.2 Phantoms

**FIGURE 5:**
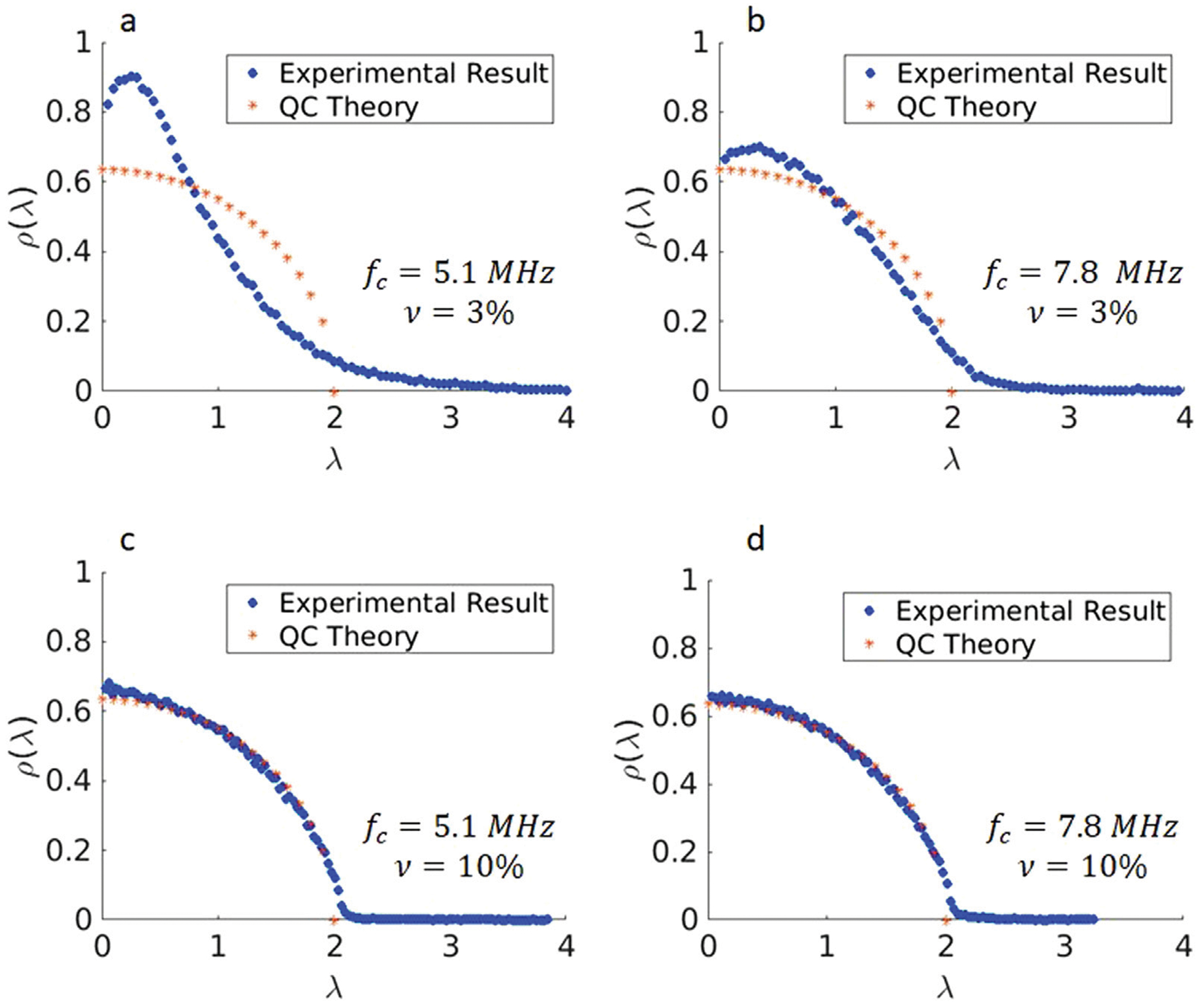
Singular value distributions of the IRM from Gelatin Glass Bead phantoms at different frequencies. **(a)**: 3% scatterer volume fraction, central frequency of 5.1 MHz **(b)**: 3% scatterer volume fraction, central frequency of 7.8 MHz **(c)**: 10% scatterer volume fraction, central frequency of 5.1 MHz **(d)**: 10% scatterer volume fraction, central frequency of 7.8 MHz.

**FIGURE 6:**
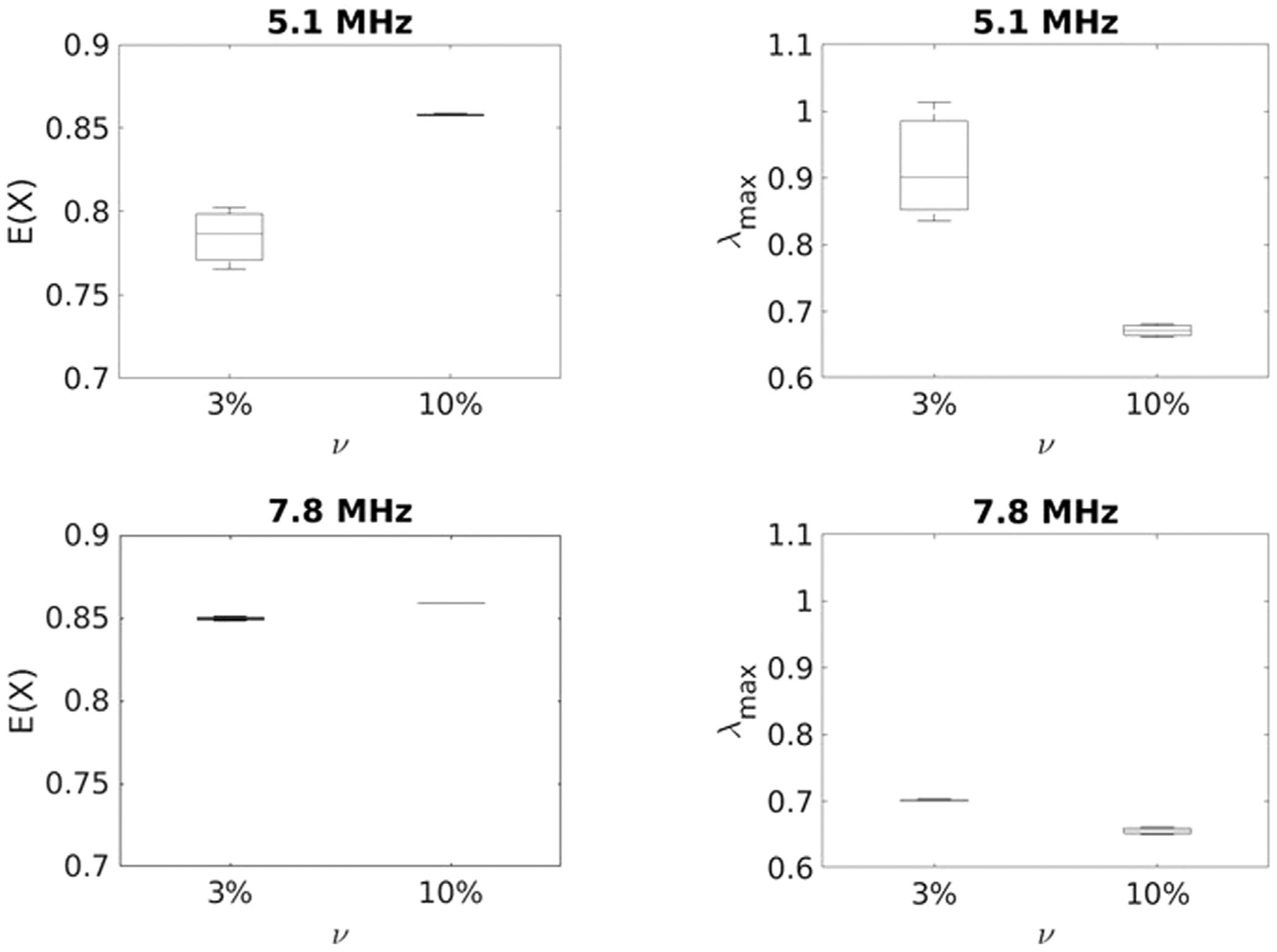
$\lambda_{\max}$ and *E*(*X*) as a function of scatterer volume fraction at different frequencies for GGB phantoms (highly scattering media).

The IRMs acquired experimentally using the L7-4 and L11-5 array transducers on the GGB phantoms were processed and the PDF for SVD was estimated for different frequencies $\left(f_{c}\right)$ and scatterer volume fractions (ν), as shown in Figure 5. For each case, the PDF of singular values was compared with the QCL from Equation 6 to assess the extent of multiple scattering. The results shown in Figure 5 imply that for the higher frequency and the higher scatterer volume fraction, the PDF follows the QCL more and more closely, which is consistent with the general results of RMT. From the physical point of view, as the frequency and scatterer volume fraction increase, multiple scattering becomes more dominant, and the randomness of the backscattered signals intensifies, which is consistent with the fact that the PDF is following the QCL law. For different frequencies, $\lambda_{\max}$ and *E*(*X*) were estimated from the PDFs. Figure 6 illustrates these values at different frequencies and different scatterer (glass beads) volume fractions. Error bars are associated with three different IRM measurements for each GGB phantoms.

The results indicate that at lower frequency both $\lambda_{\max}$ and *E*(*X*) are capable of differentiating between the two scatterer volume fractions studied here. Assuming that the gelatin has the same speed of sound as water at $f_{c}=5.1MHz$, one can show that *kr* = 2.10 where *k* and *r* define the wave number and average radius of glass beads particles respectively. However as the frequency increases, multiple scattering becomes more dominant for both 3% and 10% volume fractions, and the sensitivity of $\lambda_{\max}$ and *E*(*X*) to changes in volume fraction decreases. At the frequency of *f_c_* = 7.8 *MHz*, *kr* = 3.18. In other words, the lower *kr* the better the separation between low and high scatterer density can be achieved.

**FIGURE 7:**
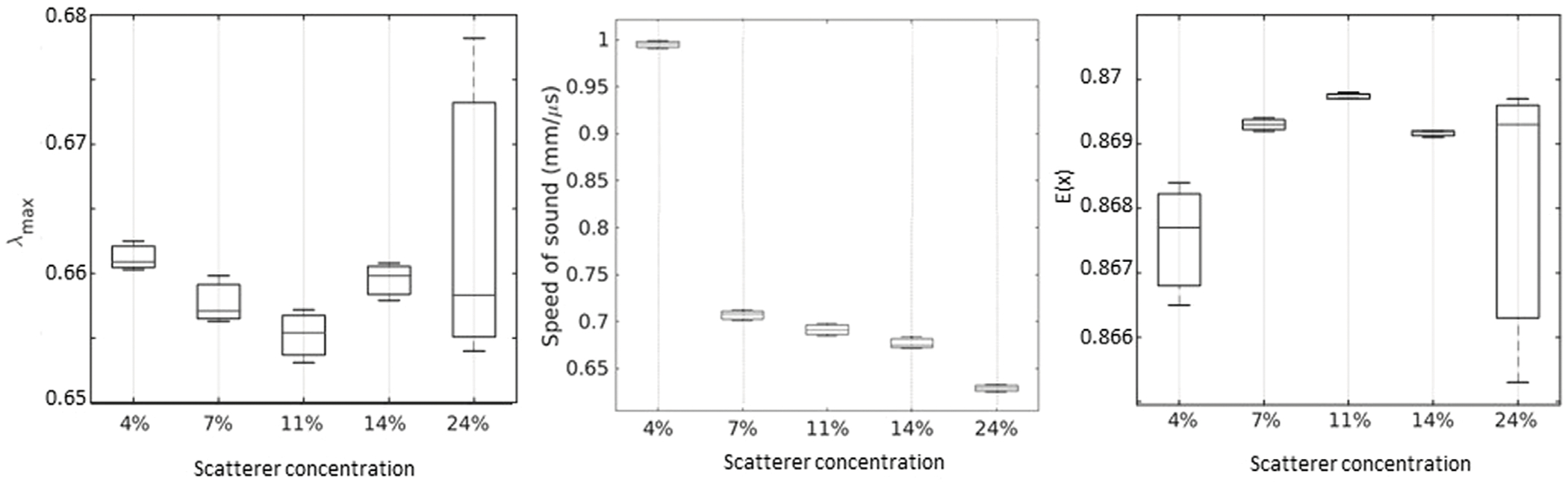
$\lambda_{\max}$, speed of sound and *E*(*X*) measured for different particle volume fractions in PDMS phantoms.

$\lambda_{\max}$ and *E*(*X*) were also estimated for the PDMS phantoms at *f_c_* = 5.1 *MHz*. Then the speed of sound was measured for three samples extracted from the phantoms. The results are shown in Figure 7.

As the figures depict, *E*(*X*) and $\lambda_{\max}$ can only detect changes for the lower volume fraction of particles studied here, corresponding to higher speed of sound values. The error bars in Figure 7 for $\lambda_{\max}$ corresponds to three different IRM acquisition locations on the phantoms. For speed of sound, the error bars correspond to three different samples extracted from each original phantom.

### 3.3 *In vivo* rodent lungs

Similarly, the IRMs acquired from the six healthy rat lungs (control group), eighteen fibrotic lungs (with varying degrees of severity), and nine rats treated with Nintedanib were post-processed to form the singular values probability distribution function (PDF). *E*(*X*) and $\lambda_{\max}$ were calculated for each IRM. For each lung, 5 IRMs were acquired to ensure the reproducibility of the data. The reported *E*(*X*) and $\lambda_{\max}$ values are the average of the parameters for all five data sets.

**FIGURE 8:**
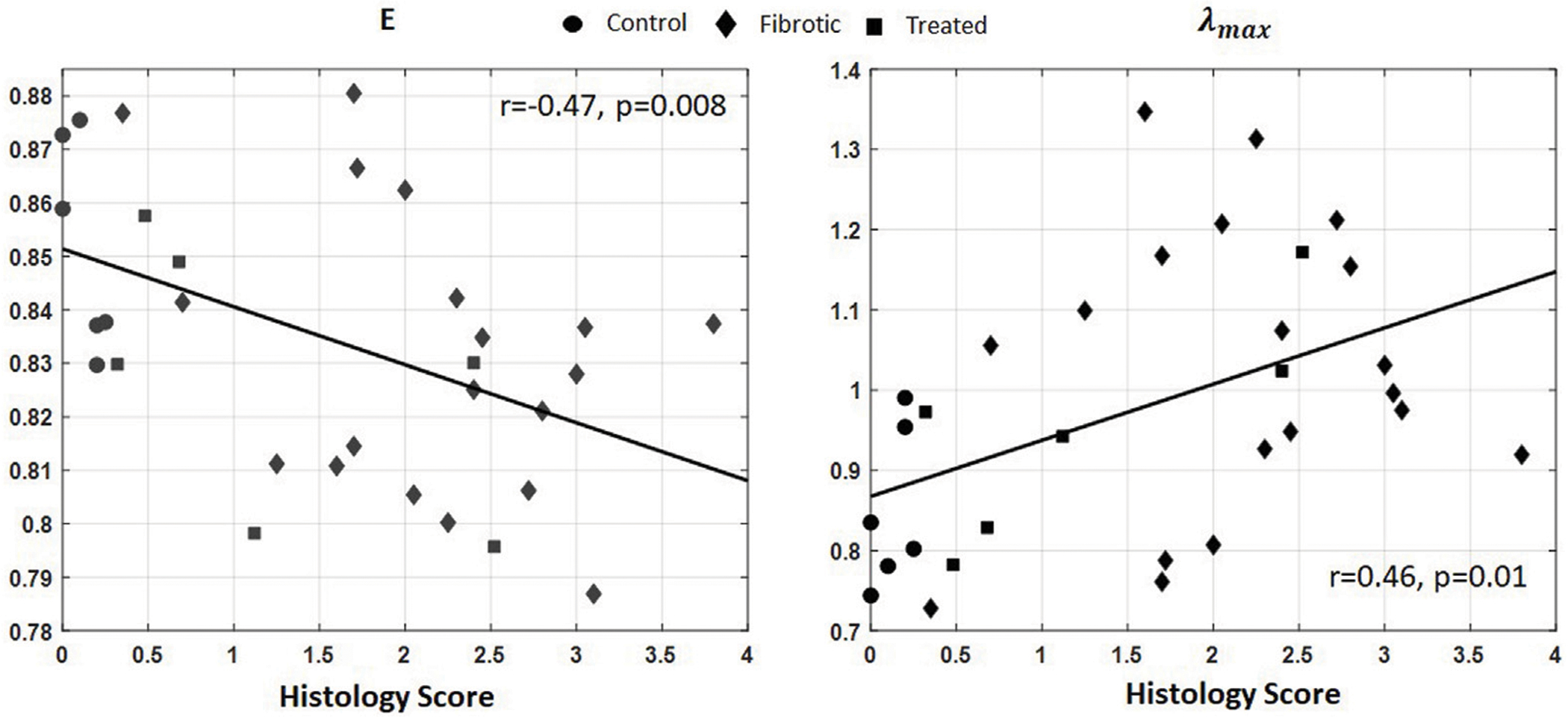
*E*(*X*) and $\lambda_{\max}$ as a function of histology score. Solid lines represent linear fits. Significant correlation with histology scores are obtained for both parameters (*p* = 0.008 for *E*(*X*) and *p* = 0.01 for $\lambda_{\max}$).

Figure 8 shows linear regressions between *E*(*X*) and $\lambda_{\max}$ and histology scores. Significant correlations with the severity of fibrosis were obtained for both *E*(*X*) (*r* = −0.47, *p* = 0.008) and $\lambda_{\max}$ (*r* = 0.46, *p* = 0.01). We also investigated the effect of time window duration on correlations of *E*(*X*) and $\lambda_{\max}$ and histology score. We examined the results for the time window duration of 2*T*, 4*T*, 6*T*, and 8*T*. No significant differences were observed between the distributions of *E*(*X*) and $\lambda_{\max}$ for various time windows.

## 4 Discussion and conclusion

In this work we used the propagation matrix in a backscattering configuration to characterize weakly and highly scattering random media using ultrasonic waves in the MHZ range. To do so, we used general results of the Random Matrix Theory (RMT) and applied them to distribution of singular values estimated from the Inter-element Response Matrix (IRM). The main goal was to investigate how changes in micro-structural parameters of a random complex medium might affect the distribution of singular values, and if, consequently, metrics of the PDF of singular values could be used to characterized complex media. The micro-structural parameter we studied was scatterer volume fraction and the metrics associated with changes in the distribution of singular values were $\lambda_{\max}$ and *E*(*X*).

We tested this hypothesis by generating 2D numerical models of highly scattering media consisting of a fluid phase (as the matrix) and solid phase (as the scatterers) for various scatterer area fractions, forming and post-processing IRM and estimating $\lambda_{\max}$ and *E*(*X*) from distribution of singular values. The numerical results indicated that as the scatterer area fraction increases, $\lambda_{\max}$ and *E*(*X*) decrease and increase respectively until they both reach a plateau. This can be attributed to the fact that multiple scattering becomes dominant after the scatterer density reaches 20% area fraction. In other words when multiple scattering is dominant over single scattering, the distribution of singular values will no longer change significantly. This is consistent with the theoretical results obtained from Quarter Circle Law (QCL) (Tulino and Verdú, 2004). Once the distribution reaches a quarter circle, it no longer changes when scatterer density increases, and is a limitation of the proposed method. The results also indicated that the values of $\lambda_{\max}$ and *E*(*X*) are sensitive to the difference in material properties of scatterers. However, it can be observed that the plateau is reached at similar scatterer area fractions for both the plastic and aluminum scatterers, suggesting that, at least in the case of these two media, scatterer density has a greater impact on the dominance of multiple scattering than scatterer impedance. This likely would not be the case for softer scatterers, with a lower impedance contrast.

As a next step, we experimentally studied how the scatterer volume fraction affects $\lambda_{\max}$ and *E*(*X*) in Gelatin-Glass Beads (GGB) phantoms mimicking highly scattering media. GGB phantoms with two different volume fractions of glass beads (3 and 10 percentage) were fabricated and using two multi-elements transducer arrays (*f_c_* = 5.1, 7.8 *MHz*) the distribution of singular values was estimated from the IRM. The results showed that at lower frequency (*f_c_* = 5.1 *MHz*), both $\lambda_{\max}$ and *E*(*X*) are capable of differentiating low from high scatterer densities. However, at the higher frequency used in this experiment, the difference is not as strong, and might not be significant. This again could be due to the fact that at higher frequency multiple scattering becomes dominant. Studying the Probability Density Function (PDF) of singular values and comparing them with QCL, we identified changes in the relative contributions of single and multiple scattering for different scatterer volume fractions, and showed that these metrics can be used in some cases to assess changes in scatterer volume fraction. Only two volume fractions were studied here. A more detailed study on the sensitivity of these metrics to scatterer volume fraction could be the subject of subsequent work. However, the true validation of these methods will only come from studies in actual biological tissues, preferably *in vivo*. The phantom study presented here was simply used as a means to prove a concept.

We also studied how $\lambda_{\max}$ and *E*(*X*) might change in more weakly scattering media. For this purpose, we fabricated PDMS phantoms with various amounts of Barium-Titanate particles to change the speed of sound in different phantoms. The results show that *E*(*X*) and $\lambda_{\max}$ are capable of differentiating between weakly scattering media only at lower scatterer concentrations. For higher concentrations of particles, the trends of *E*(*X*) and $\lambda_{\max}$ seem to change, and it becomes difficult to estimate them. The significant changes in speed of sound could offer a clue as to what is observed here. In these PDMS phantoms, changes in speed of sound were able to track the different concentrations of scatterers better than *E*(*X*) and $\lambda_{\max}$ at higher scatterer volume fraction. However, it is often difficult to measure the speed of sound, especially in biological media where through transmission configurations cannot always be achieved.

In order to determine the feasibility of such methods in biological media, we investigated whether *E*(*X*) and $\lambda_{\max}$ would reflect the severity of pulmonary fibrosis in an *in vivo* rodent model of fibrotic lungs with various degrees of fibrosis severity. A negative and significant correlation was found between *E*(*X*) and the histology score, and a positive and significant correlation was found between $\lambda_{\max}$ and the histology score. These results are consistent with the results obtained in highly scattering models *in silico* and in highly scattering GGB phantoms. The higher the histology score, the lower the density of healthy air-filled alveoli (Hubner et al., 2008), which behave as strong scatterers. *E*(*X*) is therefore expected to decrease with an increase of severity, while $\lambda_{\max}$ is expected to increase with an increase of severity. The results show the potential of these two metrics to detect and quantify the severity of pulmonary fibrosis.

## Figures and Tables

**TABLE 1:** Material properties of the simulation phases.

	Plastic properties	Aluminum properties	Water properties
Wave Speed (*mm/μs*)	2.53	6.32	1.50
Density *ρ* (g/mL)	1.21	2.70	1.00
*C*_11_ (GPa)	7.75	108	2.25
*C*_22_ (GPa)	7.75	108	2.25
*C*_12_ (GPa)	5.51	27	2.25

**TABLE 2:** Simulation parameters.

Spatial grid step	20μm	Temporal grid step	0.022μs
Simulation length	20μs	Boundary condition	Perfectly Matched Layer (PML)
Element size	300μm	Structure dimensions	40*X*20 *mm*

**TABLE 3:** Ultrasound probe parameters.

Probe	L7-4	L11-5v
Central frequency (MHz)	5.2	7.8
Element size (mm)	0.298	0.3

## Data Availability

The raw data supporting the conclusions of this article will be made available by the authors, without undue reservation.
